# Epithelial–Mesenchymal Transition Associated with Head and Neck Squamous Cell Carcinomas: A Review

**DOI:** 10.3390/cancers13123027

**Published:** 2021-06-17

**Authors:** Rogelio González-González, Gamaliel Ortiz-Sarabia, Nelly Molina-Frechero, José Manuel Salas-Pacheco, Sergio Manuel Salas-Pacheco, Jesús Lavalle-Carrasco, Sandra López-Verdín, Omar Tremillo-Maldonado, Ronell Bologna-Molina

**Affiliations:** 1 Department of Research, School of Dentistry, Universidad Juárez del Estado de Durango, Durango 34000, Mexico; rogelio.gonzalez@ujed.mx (R.G.-G.); faod@ujed.mx (G.O.-S.); omar.tremillo@ujed.mx (O.T.-M.); 2 Xochimilco Unit, Department of Health Care, Universidad Autónoma Metropolitana (UAM) Xochimilco, Mexico City 04960, Mexico; nmolina@correo.xoc.uam.mx (N.M.-F.); 2192800563@alumnos.xoc.uam.mx (J.L.-C.); 3 Scientific Research Institute, Universidad Juárez del Estado de Durango, Avenida Universidad S/N, Durango 34000, Mexico; jose.salas@ujed.mx (J.M.S.-P.); phc.smsp@gmail.com (S.M.S.-P.); 4 Health Science Center, Dentistry Research Institute, Universidad de Guadalajara, Guadalajara 4430, Mexico; sandra.lverdin@academicos.udg.mx; 5 Molecular Pathology Area, School of Dentistry, Universidad de la República, Montevideo 11600, Uruguay

**Keywords:** epithelial–mesenchymal transition, head and neck squamous cell carcinoma, EMT transcription factors, viral infections, inhibition

## Abstract

**Simple Summary:**

Mesenchymal conversion occurring in malignant epithelial neoplasms is undesirable in tumors since it promotes more aggressive tumor behavior. This phenomenon is not exclusive to head and neck carcinomas, and it is likely to be found in most neoplasms, as carcinomas are frequently aggressive. Mesenchymal conversion depends on different molecular interactions, signaling pathways, and tumor microenvironments that are related to the activation of several growth factors and diverse matrix metalloproteinases that promote ideal environments for the progression of tumor cells that are primarily associated with metastasis. This manuscript aims to review the interactions of the main molecules related to EMT.

**Abstract:**

Head and neck squamous cell carcinomas (HNSCCs) are aggressive, recurrent, and metastatic neoplasms with a high occurrence around the world and can lead to death when not treated appropriately. Several molecules and signaling pathways are involved in the malignant conversion process. Epithelial–mesenchymal transition (EMT) has been described in HNSCCs, a major type of aggressive carcinoma. EMT describes the development of epithelial cells into mesenchymal cells, which depends on several molecular interactions and signaling pathways that facilitate mesenchymal conversion. This is related to interactions with the microenvironment of the tumor, hypoxia, growth factors, matrix metalloproteinases, and the presence of viral infections. In this review, we focus on the main molecules related to EMT, their interactions with the tumor microenvironment, plasticity phenomena, epigenetic regulation, hypoxia, inflammation, their relationship with immune cells, and the inhibition of EMT in the context of HNSCCs.

## 1. Introductory Comments Related to HNSCC and EMT Phenomena

Head and neck squamous cell carcinomas (HNSCCs) are particularly aggressive neoplasms with a poor prognosis due to their high rates of local recurrence and metastasis. Approximately 850,000–900,000 cases of this epithelial neoplasm are diagnosed worldwide each year, causing an average of 450,000 deaths per year [1]. The most strongly associated risk factors are alcohol and tobacco intake, viral infections (human papillomavirus and Epstein–Barr virus), and diverse genetic factors [2,3,4].

The EMT phenomenon describes the development of nonmobile polarized epithelial cells into fibroblast-like mesenchymal cells with a great migratory ability, in which several molecular complexes and reversible processes are involved. EMT is defined as cell regulatory events that are related to a phenotypic transformation of epithelial cells into mesenchymal cells, characterized by changes in apicobasal polarity, mobility, and cell adhesion, which provide the modified cell with a greater ability for migration, invasion, and distant colonization. It is also characterized by the alteration of epithelium-specific adhesion proteins and the induction of mesenchymal proteins, as well as the overexpression of matrix metalloproteinases (MMPs) in the tumor microenvironment [5,6]. Several oncogenic pathways, the induction of hypoxia, and viral infection play significant roles in EMT progression through the activation of several transcription factors (EMT-TFs), such as Snail, Slug, Twist, and other molecules related to EMT-TFs [7]. The plasticity phenomena, inflammatory response, and epigenetic regulation in EMT have also been described, which have an important role in the development of this phenomenon.

## 2. Snail, Slug, Twist, and ZEB Are Transcription Factors Related to EMT Induction

**Figure 1:**
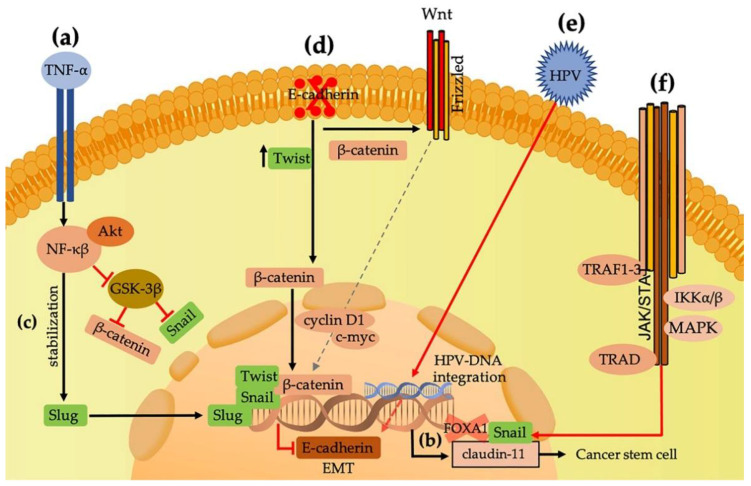
(**a**) TNF-⍺ increases the expression of Snail and Slug, regulates the expression of Twist, and inhibits GSK-3β through the phosphorylation of NF-κβ and Akt signaling pathways that induce the stabilization of Slug and β-catenin, promoting the induction of epithelial–mesenchymal transition (EMT). (**b**) The overregulation of Snail in conjunction with claudin-11 is related to the promotion of cancer stem cells (CSC) and circulating tumor cells (CTC). (**c**) The stabilization and overregulation of Slug are related to the inhibition of E-cadherin. (**d**) The low expression of E-cadherin induces the cytoplasmic overexpression of Twist and release of β-catenin to the cytoplasm, WNT canonic pathway activation, nuclear translocation of β-catenin and c-myc, and cyclin D1 amplification; the nuclear expression of Twist induces the loss of E-cadherin. (**e**) Viral HPV particles promote EMT by inducing the overexpression of Snail, Slug, and Twist and promoting the low expression of E-cadherin. (**f**) Latent membrane protein 1 (LMP1) is related to several signaling pathways that promote EMT via the induction of Snail and inhibition of E-cadherin, which is also related to the overexpression of Twist and inhibition of FOXA1-associated promotion of EMT.

EMT is promoted by diverse transcription factors, but Snail, Slug, and Twist are the most frequently reported regarding this phenomenon and directly bind to sequences in the promotor region of CDH1, which leads to the suppression of the transcription of E-cadherin [8,9]. Snail is considered an important transcription factor related to EMT induction by suppressing the transcription of E-cadherin and upregulating mesenchymal markers [10]. The expression of Snail is governed by a well-regulated signaling network in which integrin-linked kinase (ILK); phosphatidylinositol 3-kinase (PI3-K); mitogen-activated protein kinases (MAPKs); nuclear factor kβ (NFkβ); and growth factors, such as fibroblastic growth factor (FGF) and epidermal growth factor (EGF), are involved and prevent the degradation of Snail by suppressing glycogen synthase kinase 3β (GSK-3β) [11,12,13]. The upregulation of Snail in HNSCCs can induce a fibroblastic and invasive phenotype. Moreover, this phenomenon is related to the promotion of cancer stem cells (CSCs) and promotes the formation of circulating tumor cells (CTCs) through the participation of claudin-11; therefore, the overexpression of Snail and claudin-11 is related to tumor progression, recurrence, metastasis, and poor prognosis for HNSCCs [10,14,15] (Figure 1a,b). A study by Li et al. established a relationship between the upregulation of CLDN11 and SNAI1 with lymph node metastases and recurrence [15]. Snail is an important EMT-TF that can result in poor prognosis in association with recurrence and metastasis through the interaction of several signaling pathways and growth factors.

**Figure 2:**
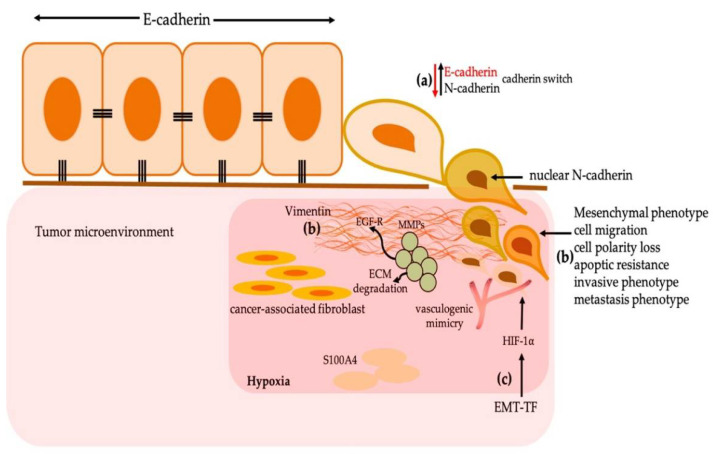
(**a**) Cadherin switch: this phenomenon is related to the gain of mesenchymal cadherins (N-cadherin) and loss of E-cadherin, which are related to the loss of cell adhesion. (**b**) The overregulation of Vimentin and N-cadherin are related to the loss of E-cadherin, induction of EMT, cell migration, loss of cell polarity, resistance to apoptosis, and invasive and metastatic phenotypes. (**c**) Tumoral hypoxia (HIF1-⍺) in the induction of EMT through epithelial–mesenchymal transition. This interaction induces vasculogenic mimicry, increases cell plasticity, promotes metastasis, and is capable of interacting with matrix metalloproteinases (MMPs), related to the degradation of E-cadherin through epidermal growth factor receptor (EGF-R), an increase in EMT-TF, the promotion of cell invasion, degradation of the extracellular matrix (ECM), tumor recurrence, metastasis, and the development of secondary primary cancers.

Similarly to Snail, Slug is an EMT-TF that is regulated by diverse growth factors, signaling pathways, and transcription factors, including Snail. Slug, a zinc finger-containing transcription factor, is encoded by SLUG and has been identified as important in the progression of cancer through E-cadherin inhibition (Figure 1c). Its upregulation contributes to EMT, tumor progression, and metastasis [16]. Zhang et al. demonstrated in HNSCCs that Slug is negatively correlated with E-cadherin expression and can induce a cadherin switch [17] that is defined by a decrease in E-cadherin and increase in mesenchymal cadherins, such as N-cadherin (Figure 2a). Katafiasz et al. found an important reduction in E-cadherin in the border cells of UM-SCC-38 (squamous cell carcinoma of the oropharynx cell culture) induced by Slug that also induced the overexpression of N-cadherin. The authors hypothesized that the overexpression of N-cadherin replaces E-cadherin in the borders of the cells. Furthermore, they indicated that Slug expression in HNSCC can induce the loss of desmosome adhesion and the modification of desmosome assembly via the loss of the expression of desmoplakin. Slug can also bind to E-box consensus sequences in the E-cadherin promoter and represses E-cadherin at the transcriptional level, inducing EMT [18]. Zinc Finger E-Box Binding Homeobox 1 and 2 (ZEB1 and ZEB2), which are members of the transcription factor family, are essential in embryonic development and tumor progression [19,20]. Both are transcriptional repressors that contain two groups of CH2H2-type zinc fingers that mediate their bond with paired CAGGTA/GE-box promotors. These repressors may induce EMT through the suppression of E-cadherin; moreover, both are related to drug resistance and cancer stem cell properties in head and neck cancer [21].

TNF-α is a pleiotropic cytokine that is induced by several inflammatory cells, especially macrophages and monocytes. It is important to highlight the association of inflammation with cancer [22]. Cancer-related inflammation is a key component of tumors and may represent the seventh hallmark of cancer. Seven hallmarks are considered in cancer, in which inflammation is included (self-sufficiency in growth signals, lack of sensitivity to anti-growth signals, inflammatory microenvironment, tissue invasion and metastasis, limitless replicative potential, sustained angiogenesis, and evasion of apoptosis) [22,23]. In the neoplastic process, inflammatory cells are important tumor promoters since they produce a favorable environment for tumor growth, facilitate genomic instability, and promote angiogenesis. Inflammatory cells, such as neutrophils, promote adhesion and tumor seeding by secreting circulating growth factors; then, platelets can interact with tumor cells and activate several signaling pathways (TNF-⍺, NF-κβ, and TGF-β/Smad) that are related to the promotion of EMT and metastasis [24,25].

TNF-α is an important key mediator of inflammation that has been associated with the promotion of tumor angiogenesis and metastasis [26]. Liu et al. evaluated the relationship among TNF-α, NF-κβ, and Slug, and they demonstrated that TNF-α increases the expression of Slug in CAL27 and HN13 (tongue squamous cell carcinoma culture cells) and prevents its ubiquitination by inhibiting its binding to GSK-3β via NFkβ [26] (Figure 1a). Slug can also regulate the C-X-C Motif Chemokine Receptor 4 (CXCR4) and C-C chemokine receptor type 7 (CCR7) proteins, related to chemokines that are overexpressed in several types of cancer, including HNSCCs, and, similarly to Twist, can induce cervical lymph node metastasis [27]. Twist is a basic helix–loop–helix protein, and its high expression has been associated with many types of cancer, including HNSCCs. Furthermore, it has been associated with cervical lymph node metastasis and, similarly to Slug, it is regulated by TNF-α and NFkβ (Figure 1a,c). Yang et al. studied the interaction between hypoxia-inducible factor 1-α (HIF1-α) and Twist, and they found that both were correlated, suggesting that hypoxia is associated with EMT [28]. As mentioned above, tobacco is an important risk factor related to the promotion of HNSCCs, mainly oral carcinomas. Zhu et al. evaluated the relationship between smoking and Twist1, and they found that Twist1 is correlated with a bad prognosis in male smokers, with a greater likelihood to acquire an EMT phenotype [29]. EMT is associated with the activation of Twist1, indicating that Twist1 is correlated with worse prognoses in male or smokers versus female or nonsmoking people, and they suggested that the expression of Twist could be modified by gender and smoking [30]. Snail, Slug, and Twist are important transcription factors whose overexpression can induce EMT. These factors are capable of inducing a loss of E-cadherin, the destabilization of cell adhesion by the modification of desmosome association, and the enhancement of mesenchymal markers associated with EMT (principally vimentin and N-cadherin). These EMT-TFs can be induced by several signaling pathways (such as NF-kβ) related to cancer, hypoxia (by the overexpression of HIF1-α), inflammation related to TNF-α, and possibly viral infection with human papillomavirus (HPV) and Epstein–Barr virus (EBV).

## 3. Complex E-cadherin/Beta-Catenin Associations with EMT

EMT is a pathological process characterized by the acquisition of changes related to the modification of epithelial phenotypes to mesenchymal phenotypes. E-cadherin is considered the main marker of the EMT phenomenon and may be the main molecule that is impaired. E-cadherin reduction leads to several alterations, such as a loss of intercellular adhesion, alterations of the basal membrane, and the induction of the nuclear translocation of β-catenin [31]. E-cadherin promotes the formation of calcium-dependent cell–cell adhesion through homophylic interactions between two molecules of cadherin on the cell surface and stabilizes adhesive attachments. In normal epithelial tissues, highly expressed E-cadherin retains β-catenin within the cell membrane, preventing its translocation from the cytoplasm to the nucleus, thereby preventing it from binding to the lymphoid enhancer-binding factor/T-cell-specific transcription factor (LEF/TCF) in the nucleus and preventing the initiation of cancer [32,33]. E-cadherin may be reduced by epigenetic alterations at several levels, including the methylation of promotors and the modification of histone proteins, as well as being modified by EMT-TFs [33,34,35]. Diverse studies have related low E-cadherin expression to the nuclear translocation of β-catenin and Wnt activation, which promotes invasion, metastasis, and poor tumor differentiation (Figure 1d) [33,36]. Steinbecher et al. observed that the expression of E-cadherin in HNSCCs was negatively correlated with Slug expression and indicated that the loss of E-cadherin is an early event in EMT [37]. Additionally, von Zeidler et al. reported the low expression of E-cadherin in the membranes of cells in high-grade oral leukoplakia and metastatic oral carcinomas [38]. De Morais et al. indicated that the cytoplasmic overexpression of Twist1 in squamous carcinomas of the lip is significantly related to the loss of E-cadherin expression in the membranes of the tumor cells, and the nuclear expression of Twist1 precipitates the loss of E-cadherin’s functions, leading to invasion (Figure 1d) [39]. β-catenin is a multifunctional protein related to the Wnt pathway and is involved in the homeostasis of tissue and embryonic development. The dysregulation of β-catenin is a critical element in tumorigenesis; it participates in the activation of canonical Wnt signaling, which plays an important role in the pathobiology of HNSCCs and promotes carcinogenesis by the amplification of diverse genes such as c-myc and cyclin D1, both of which are related to aggressive phenotypes (Figure 1d) [40,41]. In more specific terms, the aberrant expression of β-catenin is related to the promotion of cancer cell proliferation, survival, and tumor progression [42]. Therefore, a low expression of E-cadherin is associated with the nuclear translocation of β-catenin and Wnt activation, which promotes invasion, metastasis, and poor tumor differentiation related to EMT phenomena [33,36]. Studies conducted by Cercelaru et al. have found that the low immunoexpression of E-cadherin in poorly differentiated larynx carcinomas is related to the nuclear translocation and cytoplasmic overexpression of β-catenin, which could be related to the increase in Snail expression and EMT induction [36].

## 4. Vimentin and N-Cadherin (Mesenchymal Markers) Related to the Promotion of EMT

Vimentin is a type III intermediate filament that maintains cell architecture and tissue integrity. It is present in fibroblasts, endothelial cells, lymphocytes, and specialized brain cells [43,44,45]. In malignant neoplasms, its overexpression is related to tumorigenesis, EMT, and metastatic extension [43,45]. Due to its characteristics, vimentin has been considered as a predictive biomarker of tumor growth, poor tumor differentiation, and lymph node metastasis in HNSCCs. Through studies on wound healing, Cheng et al. proved that the loss of vimentin is related to a severe lack of fibroblast growth, the inhibition of TGF-β, and Slug activation [46]. Steinblicher et al. and Wangmo et al. quantified the expression of vimentin, cytokeratin, and E-cadherin and observed the overexpression of vimentin related to the low expression of E-cadherin in HSCCs [37,47]. Furthermore, it has been described that the overexpression of N-cadherin and its nuclear translocation are related to unfavorable prognoses in a diverse range of malignant tumors, including those associated with the head and neck, EMT, cell migration, cell polarity loss, apoptotic resistance, and invasive, and metastatic phenotypes [48,49,50,51]. Thus, EMT induction may be associated with the overexpression of vimentin and N-cadherin through a significant downregulation of E-cadherin and EMT-TF induction in HNSCCs (Figure 2a,b).

## 5. Plasticity Is an Important Phenomenon Related to EMT and Metastasis

The plasticity phenomenon in mammalian cells has been described as an event associated with reprogramming and induced by one or several transcription factors [52].

For example, the introduction of OSKM (OCT4, SOX2 KLF4, and MYC) may “reprogram” mammalian cells (adult human or mouse fibroblasts) to embryonic stem-like cells [53,54]. Studies have shown that several types of cells may be reprogrammed into other cells, such as cardiomyocytes and neurons [55,56,57,58,59,60,61]. In the context of EMT, the epithelial cells acquire properties of mesenchymal cells with the ability of extended self-renewal and acquisition gene expression program, similar to stem cells [62,63]. The cells with the EMT phenotype and plasticity can express epithelial and mesenchymal markers and acquired features related to cell migration, invasion, colonization, stemness, resistance to treatment, and aggressive behavior [64,65]. In HNSCC, phenotypic plasticity is related to tumor behavior, resistance to treatment, and metastasis. Studies conducted by Jian et al. showed that the growth and progression in HNSCC are related to cells with phenotypic plasticity and Paired Related Homeobox 1 (PRRX1) expression associated with EMT. The migration and invasion of cancer cells and the suppression of PRRX1 are related to the downregulation of mesenchymal markers, such as Slug, and the upregulation of E-cadherin, related to the mesenchymal–epithelial transition (MET) [66]. HNSCC cells can transition into two different stages of EMT: (1) the fixed stage, in which cells cannot return to an epithelial state, (2) and the plastic stage, in which the cells can transition to spontaneous MET and return to the epithelial phenotype [67,68]. Brabletz et al. describe that the combination of these two stages (binary characteristics, EMT and MET) provides an important mechanism of metastasis in which the tumor cells with the EMT phenotype can migrate away from the primary tumor and then, under the MET phenotype, enable the growth of a new tumor and a secondary tumor [69]. An intermediate phenomenon of EMT, known as partial EMT (pEMT), is capable of enhancing the plasticity of tumors and improving the progression of EMT and MET processes [70]. The pEMT is characterized to maintain the expression of epithelial markers and the sole expression of Slug, which is the first EMT-TF upregulated in EMT phenomena [71]. This characteristic has allowed the possibility of an intermediate stage of EMT, known as pEMT, in which the cells act similarly to cancer cells with mesenchymal characteristics, but without completely losing their epithelial features [72]. The pEMT phenomenon is characterized by high metastatic risk in comparison to the complete EMT. Kisoda et al. studied several genes related to pEMT in primary tumors of HNSCC and concluded that pEMT is related to poor prognosis [73]. Therefore, it is likely that the plasticity of pEMT (due to the binary characteristics) enhances the aggressive behavior in HNSCC.

## 6. Human Papillomaviruses and Their Influence on EMT

**Figure 3:**
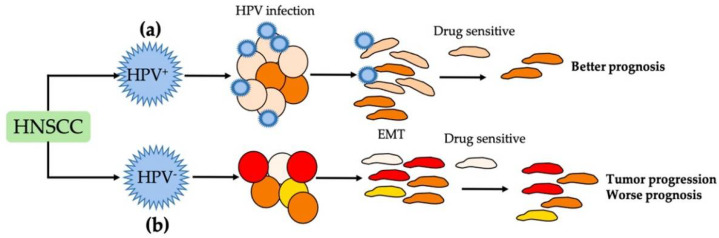
(**a**) HNSCC cells infected with HPV induce tumor homogeneity associated with sensitivity of treatment and better prognosis. (**b**) HNSCCs not infected with HPV show tumor heterogeneity related to treatment resistance and worse prognosis.

HPV is a DNA virus that shows high affinity to the stratified squamous epithelium of the mucous membrane and skin. Several studies have suggested that HPV is the main ethological virus related to the development of squamous carcinomas, and 15–35% of HNSCCs are associated with high-risk HPV, particularly HPV-16 [74]. The participation of HPV-16 in EMT is related to the capability of the viral antigens of modulating the signaling pathway of EMT and regulating the expression of E-cadherin, where a reduction in the infected squamous epithelium is shown in association with the depletion of the Langerhans cells at the site of the infection [74,75]. Cho et al. evaluated the immunohistochemical expression of Snail, Slug, and Twist1 in oropharyngeal squamous cell carcinomas (OPSCCs) and HPV-positive and -negative HNSCCs, observing elevated nuclear expression in the OPSCCs and HNSCCs. HPV-positive tumors have been shown to present the highest expression of Snail, Slug, and Twist1, which indicates that the virus’ presence increases metastatic ability by upregulating the expression of these proteins, in comparison to that observed in HPV-negative tumors [76]. (Figure 1e) Through studies performed in OSCCs, Wushou et al. observed that the overexpression of Twist, Snail, and Slug according to immunohistochemistry was present in tumors with lymph node metastasis, those in advanced clinical stages (III + IV), and moderately and poorly differentiated histological grades, along with the inhibition of the E-cadherin gene [77]. It is possible that the interaction among Snail, Slug, Twist1, and HPV-16 promotes the low expression of E-cadherin and also promotes EMT, related to poor prognosis in HNSCCs, especially in OPSCCs, which are most likely to present infection (Figure 1e). Hatakeyama et al. reported a better prognosis in patients with an OPSCC and a low expression of E-cadherin, indicating that the behaviors of OPSCCs are different from those of carcinomas located in other sites of the head and neck region, suggesting that an intratumor estimation of heterogeneity system should be developed for HNSCCs associated with EMT to inform decisions regarding the treatment of these tumors [75]. Mroz et al. and Rocco indicate the importance of studying genetic heterogeneity by showing, through next-generation sequencing data, that tumors with high heterogeneity are associated with tumor progression and poor prognosis. Moreover, they reported that HPV-positive tumors have a greater intratumor homogeneity than HPV-negative tumors, and this difference may be associated with a favorable clinical response [78,79,80]. Studies conducted by Rocco and Kagohara et al. indicate that the tumoral heterogeneity can induce different changes associated with targeted therapy resistance and poor prognosis in HNSCC (Figure 3a,b) [80,81]. According to Hatakeyama et al., HPV-positive tumors tend to lose the epithelial phenotype but tend to be homogenous (intratumoral homogeneity) and, paradoxically, have a favorable outcome, suggesting that an intratumor estimation of the heterogeneity system should be developed for HNSCCs associated with EMT to inform appropriate decisions regarding the treatment of these tumors [75].

## 7. Epstein–Barr Virus Induces EMT in Nasopharyngeal Carcinomas through Latent Membrane Protein-1

EBV was the first virus related to multiple types of cancer and their oncogenesis. In head and neck cancer, EBV is closely related to nasopharyngeal carcinomas (NPCs) [82]. Latent membrane protein 1 (LMP1) is the main oncoprotein of EBV, related to complex and related pathways, such as IKK-α, IKK-β, NF-β, SEK/JNK/c-Jun/AP-1, JAK3/STAT, interferon regulatory factor 7 (IRF7), NFkβ, and p38 mitogen-activated protein kinase (MAPK), which contribute to the positive autoregulation of LMP1 expression and EMT induction through Snail and the reduction in E-cadherin, thereby promoting early metastasis for this tumor [82,83]. (Figure 1f) A study by Ye et al. in CNE2 (nasopharyngeal carcinoma, cultured cells) found that calreticulin (CRT) expression is related to EMT by Neuropilin-1 (NRP1) expression via TGF-β/SMAD3, related to the downregulation of E-cadherin and the upregulation of vimentin, inducing EMT via SMAD3 and the TGF-β pathway [84]. LMP1 can induce the downregulation of FOXA1 and promote EMT through the overexpression of Twist1. (Figure 1f) FOXA1 is highly expressed in normal nasopharyngeal tissue and is downregulated in NPCs, and its overexpression can inhibit cell proliferation and invasion. This possibly occurs due to its ability to disrupt processes related to EMT, such as the inhibition of Twist1 [85,86].

## 8. DNA Methyltransferases, G9a, and N-Glycosylation Are Related to EMT

Epigenetic modifications are activated in EMT. These are related to histone protein tails and DNA promoter regions [87]. Epigenetic modifications in EMT are related to DNA methylation, which is a fundamental epigenetic modification catalyzed by DNA methyltransferases (DNMTs) [88,89,90,91]. DNMTs are enzymes for the addition methyl groups 5’carbon of the cytosine ring in the CpG site. There are reported five DNMTs (DNMT1, DNMT2, DNMT3A, DNMT3B, and DNMT3L). Three of these DNMTs (DNMT1, DNMT3A, and DNMT3B) are canonical enzymes and have catalytic activity, and the other DNMTs do not possess catalytic activity [92]. DNMT 1, DNMT3A, and DNMT3B are reported in mammalian cells, which are responsible for the maintenance of parental partners of DNA methylation (DNMT1) and establish new patterns of DNA methylation [93,94,95]. These DNMTs are related to EMT in HNSCCs as well. Studies conducted by Chen et al. on in-cell cultures reported that the expression of DNMT3B was aberrant in these carcinomas, and they found that CDH1 (E-cad) was downregulated, CDH2 (N-cad) and VIM (Vimentin) were upregulated, and the knockdown of DNMT3B was related to restoring E-cadherin by demethylation of the CDH1 5´region [96]. As previously reviewed, TGF-β plays the role of tumor promoter due to the induction of EMT and invasiveness. TGF-β interacts with SMAD proteins, in which SMAD4 is the key mediator of TGF-β signaling, and their downregulation is associated with tumor progression in HNSCC [96]. TGF-β may induce EMT and change the DNA methylation status by the upregulation of DNMTs in HNSCC. This hypothesis was established as Cardenas et al. found that TGF-β induces changes in DNA methylation in the EMT transition of ovarian cancer cells [97]. Moreover, the upregulation of DNMT3B in oral cancer is related to risks of lymph node involvement, recurrence, and shorter survival. This may be associated with IL-6, as well as the consumption of tobacco and betel quid chewing, which are related to the upregulation of DNMT3B by the dysregulation of miRNAs [98]. DNMT genes have polymorphic variants in which the heterozygous variant 149C/T of DNMT3B is associated with a risk of head and neck cancer, and the overexpression of DNMT1 has a higher risk of tumor relapse. Furthermore, the polymorphisms rs2228612 of DNMT1 and rs406193 of DNMT3B are associated with reduced survival in OSCC [99].

Euchromatic Histone Lysine Methyltransferase 2 (EHMT2/G9a) is a gene that encodes a methyltransferase that methylates lysine residues of histone H3, resulting in the recruitment of additional epigenetic regulators and the repression of transcription. It is considered a key methyltransferase responsible for the mono- and di-methylation of lysine 9 on histone H3 [100]. G9a can interact with Snail and promotes EMT. G9a is highly expressed in several types of malignant tumors, including HNSCC. It is associated with metastasis and poor prognosis and is related to cell proliferation, autophagy, hypoxia, cancer stemness, and EMT [101,102,103,104,105]. Shuli et al. described that Snail and G9a can form a complex (Snail-G9a) that is capable of binding to the E-cadherin in HN12 (tongue squamous cell carcinoma) cell lines and inducing EMT. They suggest that G9a is essential for inducing EMT by binding to Snail and TGF-β (G9a–Snail–TGF-β) [106].

E-cadherin can suffer posttranslational modifications that include phosphorylation, O-glycan modification, and N-glycan modification. The N-glycosylation of E-cadherin has an important influence on the tumor progression of HNSCC [107]. In EMT, the N-glycosylation of protein is catalyzed by the dolichylphospate N-acetylglucosamine-phosphotransferase (DPATG1), which regulates the loss of E-cadherin, the activation of the Wnt pathway, and the control of Wnt/B-catenin. The association between DPATG1 and Wnt/B-catenin induces the loss of E-cadherin in the cell membrane, inducing EMT; therefore, the overexpression of DPAGT1 is related to the loss of intercellular adhesion and downregulation of E-cadherin [108,109,110,111,112]. DPAGT1 promotes EMT in HNSCC by the N-glycosylation of E-cadherin and activation of Wnt/β-catenin. Studies conducted by Jamal et al. related the aberrant increase in the activity of the canonical Wnt pathway with the increase in β- and γ-catenin due to the great abundance of the DPAGT1 promoter, inducing EMT [112].

## 9. Hypoxia as an Important Factor Related to EMT

Hypoxia is important in tumor progression, where the tumor cells enhance glycolysis, regardless of the cell oxygen levels, in which related genes contribute to glucoregulatory proteins (GRPs) and O2 regulatory proteins (ORPs). Moreover, genes induced by hypoxia, such as VEGF, interleukin 1A (IL-1A), endothelin 1, platelet-derived growth factor B (PDGFB), erythropoietin (EPO), cathepsin, growth arrest and DNA-damage-inducible alpha (GADD45A), and growth arrest and DNA damage-inducible gene 153 (GADD153), as well as the presence of the hypoxia-inducible factor (HIF), are involved in tumor progression and metastasis [113]. HIF belongs to the family of basic helix–loop–helix (bHLH) proteins, contained in Per-Arnt-Sim (PAS), which are versatile sensor and interaction modules in signal transduction proteins. This sensor detects a wide range of chemical and physical stimuli and regulates the activity of functionally diverse effector domains, including hypoxia [114,115,116,117]. The HIFs are composed of three types: HIF1-⍺, HIF2-⍺, HIF3-⍺, and Hypoxia-Inducible Aryl Hydrocarbon Receptor Nuclear Translocator (ARNT) [115,116,117,118]. HIF1-⍺ is the main protein capable of promoting EMT in HNSCC through the interaction with Slug, which is a key mediator in the hypoxia-induced mesenchymal phenotype. This EMT-TF is correlated with HIF1-⍺, and together (HIF1-⍺/Slug), they play an important role in cadherin switching [17]. Additionally, HIF1-⍺ can be related to Twist. Yang et al. related the activation of Twist by the expression of HIF1-⍺ and suggested that this interaction is associated with metastasis. [28]. Twist could be overexpressed under hypoxic conditions and induce a mesenchymal phenotype; this hypoxic condition is capable of stabilizing HIF1-⍺ and inducing TGF-β1, which leads to the accumulation of HIF1-⍺. This promotes EMT by hypoxia due to its capability of reducing the levels of mRNAs of E-cadherin [119,120]. Duan et al. identified an interaction between Bcl-2 and Twist1. This complex is related to the nuclear location of Twist1, increasing the tumor cell plasticity and vasculogenic mimicry, and promoting metastasis [121]. Therefore, HIF1-⍺ induces Snail, is correlated with the expression levels of Slug, promotes tumor cell plasticity, and participates in the phenomenon known as cadherin switching. It is also associated with the promotion of the expression of Twist1, which is directly related to metastasis. HIF-α can promote the transcription of Snail by the activation of PI3K/Akt signaling and the inhibition of GSK-3β through ubiquitination. In oral cancer, hypoxia can reduce the mRNA levels of E-cadherin, thereby inducing the expression of HIF-α and EMT [113]. Figure 2c shows the tumoral hypoxia (HIF1-⍺) in the induction of EMT through epithelial–mesenchymal transition.

## 10. Tumor Microenvironment and Inflammation Are Related to HNSCC Progression

The tumor microenvironment (TME) has been related to the progression of malignant neoplasms, where the tumor stroma is important due to its relationship with invasion through a relevant role in metabolism and progression [122]. The TME of HNSCC is a highly complex ecosystem, composed of cellular and noncellular components that interact to provide “tumor survival”, and is related to EMT induction. The cellular components are composed mainly of cancer-associated fibroblasts (CAFs), endothelial cells (ECs), and infiltrating immune cells (T cells, B cells, NK cells, dendritic cells, macrophages, and myeloid-derived suppressor cells) [123,124]. The noncellular components are composed of proteins of the extracellular matrix (ECM, collagen, fibronectin, elastin, laminin, and tenascin) and other components, such as pH, oxygen, fluid flow, and interstitial flux. Furthermore, the stromal cells present in the TME are capable of providing intermediate metabolites, nutrients, hormones, cytokines/chemokines, and growth factors. These elements provide the tumor cells with adequate support for their proliferation, invasion, metastasis, and survival [125,126,127]. The interaction between TME and ECM is related to tumor-promoting immune cells, inflammatory cells, and helping tumor cells to escape immune recognition [124,125,126,127,128,129]. CAFs are abundant factors in the ECM, which have a significant role in tumor progression, and are associated with poor prognosis in several types of cancers, including HNSCC. These cells are capable of producing several types of soluble tumor factors to facilitate tumor proliferation, angiogenesis, invasion, immune escape, metastasis, and resistance to treatment [130,131,132]. The soluble tumor factors are induced by tumor cells that produce several growth factors, such as the epidermal growth factor (EGF); fibroblastic growth factor (FGF); transformant growth factor-beta (TGF-β); cytokines, such as IL-4, IL-6, IL-8, and IL-10; GM-CSF; vascular endothelial growth factor (VEGF); prostaglandin E2 (PGE2); and basic fibroblast growth factor (bFGF), which are involved in the pathogenesis of HNSCC and provide several tumor properties (inhibition of apoptosis, inflammation regulation, angiogenesis, and metastasis) related to prognosis [133,134,135,136,137]. It is important to highlight that cancer-related inflammation is an essential process in malignant diseases as it is mainly related to promoting pathways and possibly initiating cancer. Oncogenes (myc, ras, and ret) can lead to the constitutive production of inflammatory cytokines by the initiated tumor cells [22]. CAFs and tumor cells need reciprocal communication to stimulate mediators such as vimentin, matrix metalloproteinase (MMPs); periostin; Insulin growth factor-2 (IGF2); IL-33; and CXCL12, related to and tumor growth, invasion, and downregulation of tumor suppressor genes. Furthermore, CAFs are capable of increasing tumor cell proliferation and provide some properties related to resistance to chemotherapy. CAFs are located in the vicinity of tumor cells and can enhance tumor growth by the secretion of growth factors and MMPs, and their paracrine secretion is related to tumorigenic mechanisms that aim to achieve EMT [124,132,138,139]. Cancer stem cells (CSCs) are another hallmark of cell plasticity that is present in the ECM, and together with EMT, these have been identified as dangerous due to their promotion of tumor progression, metastasis, resistance to treatment, and tumor recurrence [29]. There is no cell-autonomous effect in the interaction between EMT and CSCs, which is mediated by cell communication and intracellular signaling networks related to EMT and CSCs, in which the Notch-Jagged signaling pathway that is related to the promotion and hybrid epithelial/mesenchymal participates (E/M, considered as a phenotype of pEMT) [140]. The phenomena related to the triggering of EMT may contribute to the development of an inflammatory and immunosuppressive response.

The increase in inflammatory mediators in HNSCC is related to patients who smoke tobacco, with enhanced invasion, angiogenesis, and metastasis [141]. Inflammatory cytokines, growth factors, prostaglandins (PGE2), and interleukins (IL-1β) have been related to a progression in HNSCC. For example, IL-1β can upregulate COX-2 expression and regulate important cellular functions. Additionally, these interleukins and proinflammatory mediators upregulate ZEB1, which is a transcription factor related to EMT and can downregulate CDH1 by binding to a subset of E-boxes, resulting in a transcriptional repressor [142,143,144]. St John et al. and St John evaluated the relationship between IL-1β and HNSCC culture cells (Tu686 and Tu212), and they found that IL-1β upregulates COX-2, increases the levels of PGE2, and upregulates Snail, increasing the capability of metastasis and pro-inflammatory mediators. Finally, they suggested that IL-1β may be an autocrine or paracrine modulator of Snail in HNSCC [145,146].

It is important to highlight the presence of S100A4 in the tumor stroma of HNSCCs, which is related to invasion and metastasis, where inflammatory cells can express S100A4 and contribute to the aggressive behavior of these tumors [21]. Another important characteristic of tumor stroma is its quantity, defined as the tumor stroma ratio (TSR). Karpathiou et al. indicated that laryngeal and esophageal squamous carcinomas rich in tumor stroma are associated with poor prognosis. Moreover, they indicated that the presence of tumor budding, smaller cell nest sizes at the core area, invasive margins, and fibroblastic stroma are adverse prognostic factors [147].

## 11. Overexpression of Matrix Metalloproteinases Are Related to Tumor Progression and EMT Induction

Matrix metalloproteinases (MMPs) are multiple zinc-dependent endopeptidase families involved in several physiological functions and pathological processes, such as carcinogenesis, tumor growth, invasion, and metastasis. MMPs play a pivotal role in invasion and metastasis due to the degradation of ECM and endothelial membrane cells. An MMP with an important activity in tumor invasion is MMP9, which is expressed in several tumors, including HNSCC [148]. Zuo et al. evaluated the participation of epidermal growth factor receptor (EGFR) and MMP20, and they found that EGFR activation induces the degradation of E-cadherin, and induces EMT-like cells by ERK-1/2 and PI3K-regulated MMP9 signaling pathways [149]. Aseervatham et al. found that the expression of MMP20 is related to the increase in Vimentin, Snail, and Twist in oral squamous cell carcinoma cells. Therefore, the silencing of MMP20 is related to a decrease in EMT-TF, N-cadherin, and Vimentin [150]. Khales et al. evaluated Twist1 and MMPs in KYSE-30 (a cell line of esophagus carcinoma) and related the overexpression of Twist1 with an increase in the transcription of MMP2/3/7/9/10, finding a greater migration ability in this cell line [151]. Pietruszewka et al. found an elevated expression of MMP1 and MMP2 in immunohistochemical studies of tumors in advanced clinical stages (TNM/AJCC 3 and 4) of HNSCCs [152], similarly to De Carvalho et al. and Zhang et al., in which the overexpression of MMP9 was related to tumor recurrence, lymph node metastasis, and the development of second primary cancers [153,154]. Yan et al. indicated that the presence of MMP17 (membrane-type 4 MMP, MT4-MMP) is capable of promoting cell invasion under hypoxic conditions and observed that MMP17 promotes ameboid cell movement, invadopodia, and the degradation of the ECM in FaDu cells (a cell line of hypopharynx squamous carcinomas) [155]. Studies conducted by Huang et al. in HNSCC cells indicate that MMP17 is induced by HIF1-⍺-mediated hypoxia and enhances metastasis [156] (Figure 2c).

## 12. Inhibition of EMT Is Important in the Treatment of HNSCC

As described above, EMT-TFs and mesenchymal markers can interact with several signaling pathways associated with EMT that are related to the MMP stimulus, tumor budding, invasion, metastasis, and resistance to treatment. Different therapies have been studied for inhibiting EMT by focusing on the inhibition of signaling pathways and EMT-TFs. MicroRNAs (miRNAs) were identified in 1993 as a class of endogenous small non-coding RNAs related to the regulation of several roles through the union to mRNAs for cleavage or translational repression and with a potential function as oncogenes or tumor-suppressive genes in cancer [157,158,159]. Novel therapies with microRNAs have been proposed that are capable of inducing negative or positive interactions with EMT. In this regard, the miR-200 family acts as a tumor suppressor [160]. This family has properties related to epithelial and mesenchymal phenotypes and is closely related to EMT phenomena. In HNSCCs, miR-200a/b/c can inhibit EMT by the repression of ZEB1 and ZEB2 (transcription factors that repress E-cadherin and promote EMT in HNSCCs). The most important feature of the relationship between miR-200 and ZEB is that ZEB1 can repress the transcription of miR-200 by inducing a double-negative feedback loop, promoting EMT in HNSCCs [161,162]. A study by Kim et al. evaluated the relationship of an RNA-binding protein called quaking (QKI) related to miR-200a/b and found that the knockdown of QKI in CAL27 (tongue squamous cell carcinoma, cultured cells) promoted cancer cell growth and EMT in relation to an increase in ZEB1, vimentin, and N-cadherin. Moreover, they observed that the overexpression of miR-200 in cells induces the migratory ability induced by ZEB1, while the overexpression of QKI impairs this [162]. Therefore, the overexpression of QKI and miR-200 is capable of inhibiting EMT in HNSCCs, despite the migratory capacity induced in miR-200 by ZEB1. Another important miRNA related to the tumor microenvironment is miR-149-3p, whose overexpression has been associated with a reduction in tumor neovascularization and a decrease in fibroblast growth factor-2 (FGF-2) signaling, playing an important role in the tumor microenvironment and the reduction in hypoxia, inhibiting the proliferation of OSCC cells, inducing apoptosis via the activation of caspase 3 [163,164,165], and possibly acting against EMT. Li et al. observed that the overexpression of miR-625 is capable of inducing the inhibition of EMT by the increase in the expression of E-cadherin and the decrease in the levels of N-cadherin and Vimentin. It is also capable of blocking the sex-determining region Y-box 4 (SOX4) in HNSCC [166]. SOX4 is considered as the main regulator of EMT, which is related to the induction of tumorigenesis and metastasis [167,168]. The presence of CSCs in HNSCC has been reported in several studies that are related to tumor progression, metastasis, and treatment resistance. The presence of CSCs and Wnt/β-catenin signaling is related to the resistance to treatment due to the receptors of Wnt (frizzled relate proteins) that promote resistance in several tumors [169,170]. Secreted frizzled-related protein-4 (sFRP4) is involved in the regulation of apoptosis, proliferation, and tumor growth [171,172,173,174]. As previously described, the loss of E-cadherin releases β-catenin into the cytosol and activates the Wnt signaling pathway to promote nuclear translocation. Warrier et al. evaluated an increase in the expression of E-cadherin following sFRP4 treatment associated with the induction of MET and observed the downregulation of Twist and Snail. They observed that sFRP4, an endogenously expressed Wnt antagonist, is capable of inhibiting CSC growth [175].

Hyperthermia is a modality of treatment related to an increase in the efficacy of conventional treatment approaches, which has been adopted as a minimally invasive treatment of some metastatic tumors in different organs [176,177]. Hyperthermia is related to the inhibition of tumor growth and contributes to the enhancement of therapy against cancer. It is also related to tumor cell killing and the sensitization of these cells to radio and chemotherapy [178,179]. Tang et al. studied the effect of hyperthermia on EMT, especially with Twist2, which is associated with EMT and metastases in HNSCC. They found that hyperthermia can reduce the expression of Twist2 by decreasing the capability of cell migration and increasing the levels of mRNA of E-cadherin expression in tongue squamous cell carcinoma cells [180].

The use of isothiocyanates (ITCs), which are natural compounds found in crucifer vegetables, has been proposed. These ITCs are related to anti-proliferative and apoptotic activity, as they are capable of destabilizing the mitochondrial membrane and inducing apoptosis by the increase in Bax and the inhibition of Bcl-2 and Bcl-XL [181,182]. Ma et al. evaluated the activity of Benzyl isothiocyanate and found that this ITC is capable of promoting apoptosis by the induction of caspase-3 and inhibiting the expression of MMP9, which is related to the induction of EMT [182].

The use of propofol has been proposed, although its use is still controversial. Propofol is an intravenous short-action anesthetic in which anti-neoplastic properties have been described, associated with the inhibition of cell proliferation, invasion, and angiogenesis, and is related to the inhibition of MMPs 2 and 9 in esophagus carcinoma cells [183]. Studies conducted by Li et al. established the association of the use of propofol with the increase in Snail and the promotion of EMT in tongue squamous cell carcinoma cells [184].

## 13. Conclusions

EMT is an important phenomenon capable of inducing aggressiveness, invasion, metastasis, proliferation, recurrence, and resistance to treatment via the interaction of several molecules and tumor microenvironments. The papers analyzed in the current review provide evidence that EMT can induce several interactions between cells and stromal tumors at the intracellular and extracellular levels. These interactions provide cells with greater capabilities for mesenchymal transformation, proliferation, invasion, angiogenesis, and metastasis, and they can be enhanced by inflammation and hypoxia, conferring resistance to conventional treatment. Head and neck tumors associated with EBV and HPV may show greater potential for EMT, aggression, and resistance to treatment. In tumors infected with HPV, it is important to detect homogeneity through molecular techniques in HNSCCs to establish therapeutic strategies focused on this phenomenon. Studies have recently been conducted regarding miRNA, QKI, hyperthermia, and the use of ITC as therapeutics to inhibit EMT to reduce tumor aggressiveness and improve treatment responses. However, the use of hyperthermia, ITC requires further studies to evaluate its capacity to inhibit EMT, and the use of propofol requires further studies focusing on HNSCCs with EMT phenotypes to evaluate their efficacy in these tumors.

## Data Availability

Not applicable.
